# EAPB: entropy-aware path-based metric for ontology quality

**DOI:** 10.1186/s13326-018-0188-7

**Published:** 2018-08-10

**Authors:** Ying Shen, Daoyuan Chen, Buzhou Tang, Min Yang, Kai Lei

**Affiliations:** 1 Shenzhen Key Lab for Information Centric Networking & Blockchain Technology (ICNLAB), School of Electronics and Computer Engineering, Peking University Shenzhen Graduate School, 518055 Shenzhen, People’s Republic of China; 2 School of Computer Science and Technology, Harbin Institute of Technology (Shenzhen), 518055 Shenzhen, People’s Republic of China; 3 SIAT, Chinese Academy of Sciences, 518055 Shenzhen, People’s Republic of China

**Keywords:** Ontology evaluation, Ontology modeling, Entropy-based metric, Knowledge representation, Big data and semantics

## Abstract

**Background:**

Entropy has become increasingly popular in computer science and information theory because it can be used to measure the predictability and redundancy of knowledge bases, especially ontologies. However, current entropy applications that evaluate ontologies consider only single-point connectivity rather than path connectivity, and they assign equal weights to each entity and path.

**Results:**

We propose an **E**ntropy-**A**ware **P**ath-**B**ased (EAPB) metric for ontology quality by considering the path information between different vertices and textual information included in the path to calculate the connectivity path of the whole network and dynamic weights between different nodes. The information obtained from structure-based embedding and text-based embedding is multiplied by the connectivity matrix of the entropy computation. EAPB is analytically evaluated against the state-of-the-art criteria. We have performed empirical analysis on real-world medical ontologies and a synthetic ontology based on the following three aspects: ontology statistical information (data quantity), entropy evaluation (data quality), and a case study (ontology structure and text visualization). These aspects mutually demonstrate the reliability of the proposed metric. The experimental results show that the proposed EAPB can effectively evaluate ontologies, especially those in the medical informatics field.

**Conclusions:**

We leverage path information and textual information to enrich the network representational learning and aid in entropy computation. The analytics and assessments of semantic web can benefit from the structure information but also the text information. We believe that EAPB is helpful for managing ontology development and evaluation projects. Our results are reproducible and we will release the source code and ontology of this work after publication. (Source code and ontology: https://github.com/AnonymousResearcher1/ontologyEvaluate).

## Background

The term ontology refers to “a representation and definition of the categories, properties, and relations of the concepts, data, and entities that substantiate one, many, or all domains.” [1] Ontology has attracted increasing attention recently due to its broad applications such as information retrieval, relation extraction, and question answering. Significant progress has been made in the ontology construction [2]. However, the ontology evaluation is still a relatively new territory and under-explored. As a result, there are few commonly agreed-upon methodologies and metrics for ontology evaluation.

Considering each ontology as a graph or a network, entropy can be used as a measure of the complexity and redundancy of the graph. Ontologies may contain data and concepts redundancy that could be removed for the sake of consolidation and conciseness without changing the overall meaning. The information density is operationalized based on the normalized entropy measured between all concept pairs in the ontology [3]. States of lower entropy occur when ontology become organized. In the literature, the entropy evaluation of the lexical information included in the ontology has been studied in the last decade and have been proved to be helpful for ontology evaluation [4].

Despite the effectiveness of previous studies, current entropy applications used to evaluate ontology have three limitations, in that they (1) Exclusively consider single point connectivity rather than paths [5], which neglects information pertaining to non-adjacent nodes. (2) Assign equal weights to edges and paths [6], which induces a loss of diversity. (3) Assume vertices are static, which ignores the various aspects of vertices when interacting with different neighboring vertices [7].

To address these three limitations, this article describes an Entropy-Aware Path-Eased quality metric for ontologies (EAPB) by comparing their information densities to those of other ontologies. We consider the path information between different vertices in ontology as well as the textual information included in the path to calculate the dynamic weight between different nodes and the connectivity path of the entire network. Specifically, we first apply CNN to learn the structure-based embedding and text-based embedding to capture both the ontology network structures and their encapsulated textual information. Subsequently, the information gain which is in the form of a matrix obtained by a cosine similarity calculation of the relevancy between nodes *u* and *v*, is multiplied by the connectivity matrix of entropy computations. Finally, we validate the effectiveness and robust superiority of our model on four real-world ontologies.

Three infectious disease-relevant ontologies, i.e. Infectious Disease Ontology^1^ (IDO), Infectious Disease Ontology for Dengue^2^ (IDODEN), and Disease Ontology^3^ (DO) are adopted as baselines. Our material includes an in-house ontology that is used to develop an ontology-driven clinical decision support system for infectious disease diagnosis and antibiotic prescription (IDDAP) [8]. To demonstrate the applicability and generality of our quality metric for ontologies, we conduct evaluations on real-world ontologies with different structures and different textual information. To verify whether our quality metric can make a significant performance boost by incorporating textual information into the EAPB architecture, we assess ontologies with the same structures but different textual information, as well as report the ablation tests in terms of discarding the textual information. The textual attention visualization and ontology statistical information are used as references to evaluate the validity of the calculation.

To summarize, the core contributions of this study are as follows:

- To overcome the single point connectivity and equal weight problem, we consider the path information between different vertices in an ontology and the text information included in the path. The information gain obtained from these sources are used to adjust the connectivity matrix of entropy computation to provide a reliable metric for evaluating ontology redundancy.
- To solve the unreal assumption of traditional ontology computations, in which each vertex is represented as a static embedding vector, we consider that the nodes’ interactions are dynamic by adapting mutual attention to emphasize those words that are focused by its neighbor vertices. The neural models are beneficial for representing ontology information.
- An ontology assessment is conducted on four real-world ontologies from three aspects: ontology statistical information (data quantity), entropy evaluation (data quality), and case study (ontology structure and text visualization), which mutually reflect the reliability of the proposed quality metric. The experimental results show that, compared with existing methods, EAPB can more effectively evaluate ontologies.

The remainder of this paper is organized as follows. “Related Work” section reviews the previous state-of-the-art ontology assessment methods. “Methods” section introduces the materials, i.e., ontologies related to infectious diseases. “Experiment and Results” section proposes the EAPB quality metric with information divergence by considering both text-based and structure-based embedding. “Discussion and conclusion” section presents the ontology statistical information, the evaluation experiments, and summarizes the evaluation results. Conclusions and possibilities for future work are outlined in “Methodology” section.

## Related work

### Ontology assessment with information density and entropy

Zaveri et al. [9] unified and formalized commonly used terminologies across papers related to data quality and provided a comprehensive list of the dimensions and metrics. In this list, these authors qualitatively and quantitatively selected 18 data dimensions, including accessibility dimensions, intrinsic dimensions, contextual dimensions, and representational dimensions, involving 69 metrics. Similarly, Färber et al. [10] used 34 evaluation indicators to perform statistics, analyses and comparisons of five renowned databases: DBpedia, Freebase, OpenCyc, Wikidata, and YAGO.

Ontology metrics can be divided into three main dimensions: structural, functional, and usability-profiling. The structural dimension of ontologies exploits the syntax and formal semantics of the ontologies represented as graphs. In this form, the topological, logical and meta-logical properties of an ontology are measured by means of a context-free metric [11]. The functional dimension is related to the intended use of the context of the given ontology and its components, while the usability-profiling dimension employs the ontology annotations to address the communication context of an ontology [11]. Considering that redundancy is not only related to the subcharacteristics of structural dimension (e.g., a high structural redundancy indicates potential tangledness) but also to the subcharacteristics of the other two dimensions (e.g., a high structural redundancy indicates improper modularity, and a high textual redundancy harms the reusability since that the classes are easily confused) [12], we take into account these three metrics and conduct the entropy-based metric in deep neural network to interactively learn the structural information included in the ontology and the context information from entity annotations.

Entropy has become increasingly popular in computer science and medical informatics [13]. Calmet et al. [6] realized the distance measure using entropy and mutual information from information theory. They considered ontological structures and distances using centrality measures, such as the degree, closeness or betweenness. However, all the edges were assumed to have the same weight. Based on [6], Doran et al. [7] proposed a reformulation of the entropy metric to evaluate the amount of information carried by both the ontology structure and the semantics associated with the edges of the ontological graph. However, the assumption of [7] that all language level edges must be equal cannot be applied in most cases. Gurupur et al. [5] calculated the probability distributions and the information entropy of the knowledge base with a new metric that measures the node source’s connectivity strength based on the number of unique paths from one node to another. Nevertheless, the study of [5] exclusively considers single-point connectivity rather than multiple connected paths.

### Assessing ontology using network embedding

Network embedding aims to map vertices of a network (ontology) onto a low-dimensional space according to their structural roles in the network.

In recent years, a large number of network embedding models have been proposed to learn efficient vertex embeddings, including DeepWalk, LINE, and node2vec. However, these structure-only models do not consider the information that accompanies the vertices in networks. Therefore, compared with sophisticated deep learning architectures such as convolutional neural networks, these methods usually yield inferior results when applied to particular machine learning tasks.

Many studies have attempted to incorporate information into network embedding models or convolutional neural networks. For example, Yang et al. [14] proposed the text-associated DeepWalk model to incorporate textual features of vertices into network representation learning under a matrix factorization framework. Zhang et al. [15] proposed a content-enhanced network-based computational approach to jointly leverage the network structure and the content information. Tu et al. [16] proposed a max-margin Deep-Walk to enhance the discriminatory ability.

Most network embedding methods rely solely on network structure while ignoring the diverse roles of vertex interactions. The rich network content information used to describe the characteristics of a node is beneficial for evaluating entropy.

## Methods

### Ontologies for infectious disease

To verify the effectiveness of EAPB, four real-world ontologies related to infection diseases are adopted for comparison. Infection constitutes the invasion of an organism’s body tissues by disease-causing agents, the multiplication of these agents, the reaction of host tissues to these organisms, and the toxins produced by these organisms [17, 18].

1. Disease Ontology (DO)

The Disease Ontology (DO) database represents a comprehensive knowledge base of 8043 inherited, developmental and acquired human diseases [19]. The DO database is divided into eight categories, including diseases by infectious agent, diseases of metabolism, diseases of mental health, and five others.

2) Infectious Disease Ontology (IDO)

The IDO [20] was designed as a set of interoperable ontologies that provide coverage of the infectious disease domain. At present, it is considered the most complete infectious disease ontology. However, the IDO lacks some infectious disease-relevant classes, such as “hemolytic-uremic syndrome.”

3) Dengue ontology (IDODEN)

The IDODEN is an extension of the Infectious Disease Ontology (IDO) for dengue fever [21]. IDODEN reuses the Malaria Ontology IDOMAL [22], an existing infectious disease ontology. IDODEN contains a wide spectrum of ontological descriptions, from descriptions of the disease itself to descriptions of vector biology, virus biology, and epidemiology.

4) IDDAP ontology

For conciseness, the ontology applied in the IDDAP system we developed is called IDDAP ontology. The ontology hierarchical conceptual schema of IDDAP ontology covers the following nine dimensions: (1) disease, (2) infection site, (3) bacteria, (4) animal, (5) symptom, (6) symptom type, (7) situation, (8) complication, and (9) antibiotic. Many infectious disease-relevant ontology resources were reused to construct the IDDAP ontology, including the DO and IDO mentioned above, as well as the NCBI organismal classification ontology,^4^ the Human Phenotype Ontology,^5^ DrugBank,^6^ Antibiotic Guidelines (2015–2016),^7^ the Antibacterial Spectrum Guide, and various websites (e.g., the U.S. National Library of Medicine (NLM)^8^). For example, the species included in the NCBI (including bacteria, viruses, fungi, and animals) were adopted to facilitate the completion of the IDDAP ontology knowledge base.

**Fig. 1:**
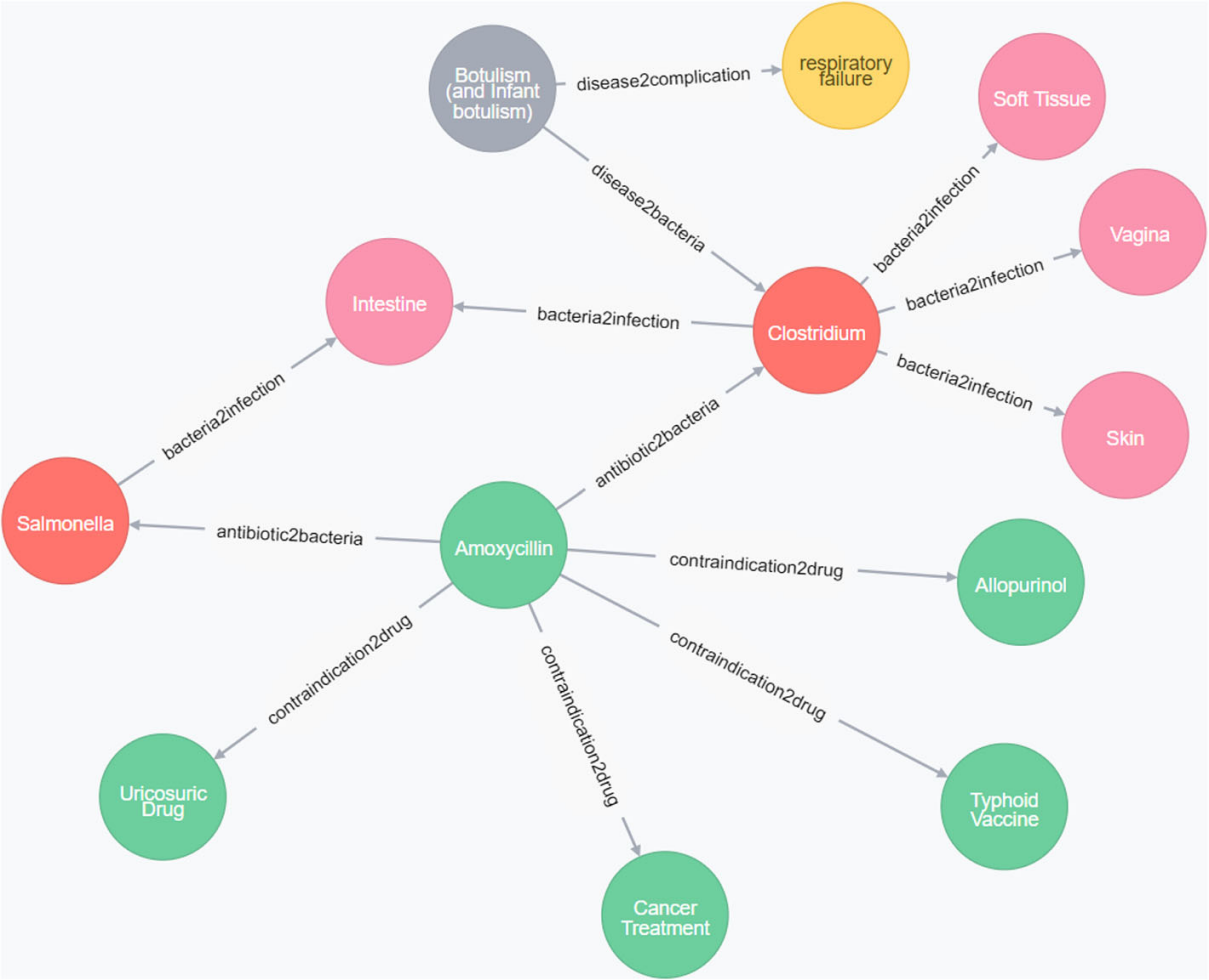
IDDAP ontology—Amoxicillin. The green nodes represent contraindications between Amoxicillin and other drugs (relation_ contraindication_drug). The red nodes identify bacteria that can be treated by Amoxicillin (relation_antibiotics_bacteria). The pink nodes indicate the relationships between infection sites and bacteria (relation_infs_bacteria). The gray nodes specify the relationships between diseases and bacteria (relation_disease_bacteria). Finally, the yellow nodes show the relationships between diseases and complications (relation_disease_ complication)

The constructed domain ontology contains 1,267,004 classes, 7,608,725 axioms, and 1,266,993 members of “SubClassOf” that pertain to infectious diseases, bacteria, syndromes, anti-bacterial drugs and other relevant components. The system includes 507 infectious diseases and their therapy methods in combination with 332 different infection sites; 936 relevant symptoms of the digestive, reproductive, neurological and other systems; 371 types of complications; 838,407 types of bacteria; 341 types of antibiotics; 1504 pairs of reaction rates (antibacterial spectrum) between antibiotics and bacteria; 431 pairs of drug interaction relationships; and 86 pairs of antibiotic-specific population contraindicated relationships. The amoxicillin class of IDDAP ontology is presented as an example (Fig. 1).

A graph G(V, E) is a math concept [23] that represents a set of vertices V and edges E. An ontology is an undirected graph with edges that connect unordered pairs of vertices. The IDDAP ontology and its three baselines (IDODEN, IDO, and DO), which contain no loops or multiple edges, can be considered as simple graphs.

### Comparison of ontologies

The studied ontologies are compared by assessing the numbers of triples, classes, entities and relationships among the IDO, IDODEN, DO and IDDAP ontologies.

#### Triples

The quantity of triples included in different ontologies is calculated.

#### Classes and Entities

The classes of IDO, IDODEN and DO are recognized via the labels owl:Class and rdfs:subClassOf, whereas the class of IDDAP is identified via Class IRI. The statistics of the class quantities included in the ontologies is conducted using Protégé.

#### Relationships

The number of relationships is calculated via the labels owl:ObjectProperty, owl:AnnotationProperty and owl:DataProperty.

#### Granularity

Ontology granularity refers to the levels of semantic detail carried by an entity and the structural abstraction of entities. In this study, we estimate the ontology granularity by counting the number of properties with the same label. For example, DBpedia has two types of relationships for the word “creator”, dbo:author and dbo:director, whereas YAGO only has only one relevant relationship, yago:created. In this case, DBpedia has finer granularity than YAGO.

## Experiment and results

The ontology assessment is conducted based on the following three aspects: ontological (statistical) information (data quantity), entropy evaluation (data quality), and case study (ontology structure and text visualization).

### Ontology information statistics

Table 1 shows the statistical information from IDO, IDODEN, DO and IDDAP. We can observe that the number of triplets and classes increases while the number of relations decreases. IDDAP has fewer relationships which are predefined, including the relationships of disease_complication, disease_symptom, disease_bacteria, and symptom_type. In Tables 1 and 3, the entries with largest values are italicized for facilitating the reader's reading.

**Table 1:** Summary statistics of ontologies

	IDO	IDODEN	DO	IDDAP
Triples with annotation/data	3901	23,657	129,670	*3,807,709*
Triples without annotation/data	960	5845	10,060	*1,272,502*
Class/Instance/Entity	507	5007	11,088	*1,267,005*
Subclassof	582	5834	10,008	*1,266,996*
Equivalent classes	81	0	*46*	*1100*
Disjoint classes	17	11	*6*	*23*
Object property	*39*	25	20	8
Annotation property	*63*	*63*	33	1

**Fig. 2:**
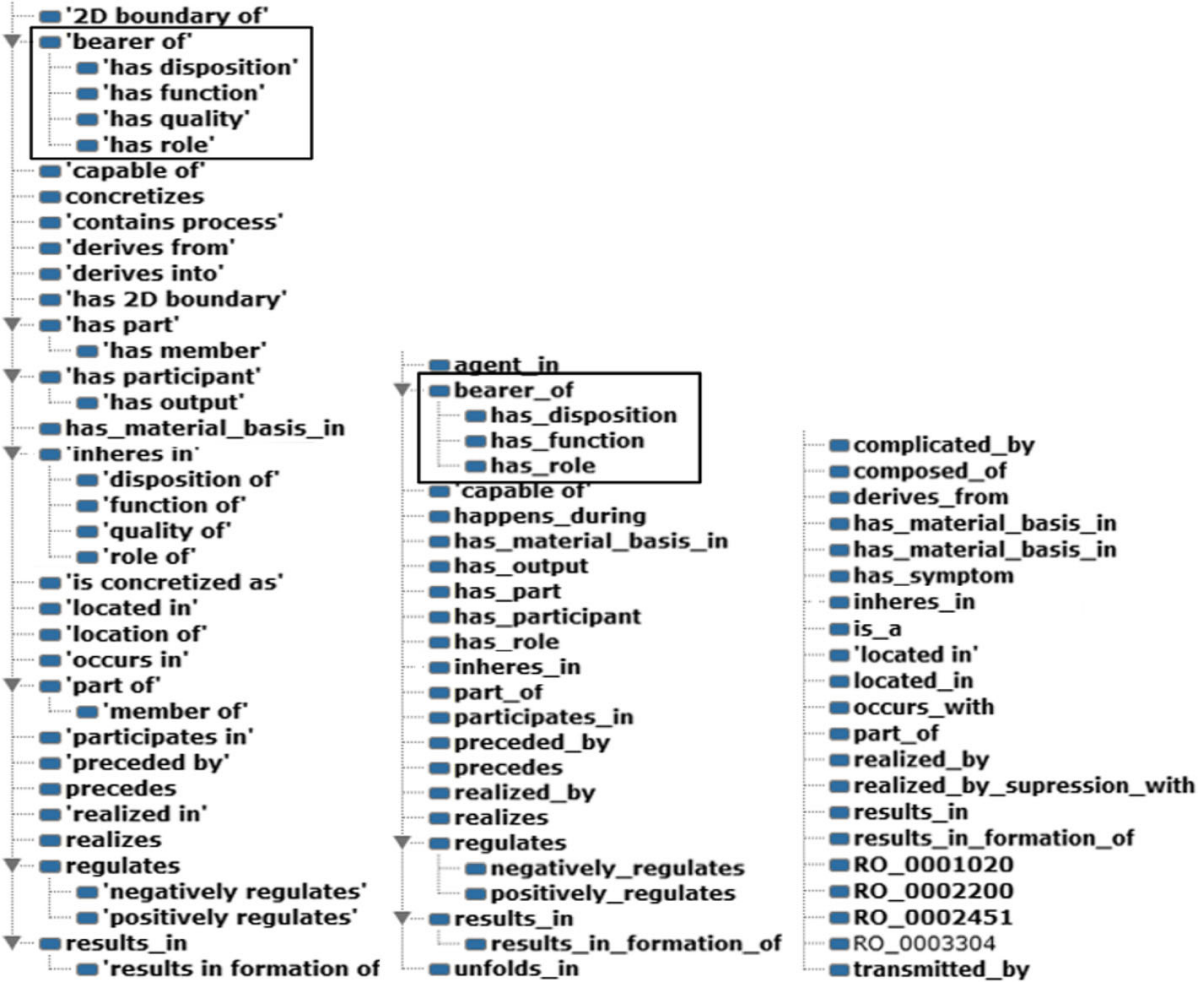
Granularity observation in the IDO, IDODEN and DO ontologies. From left to right: the granularity decreases due to the increase of sparseness of relationship types in ontology schema

With different relationship types in their schema, IDO, IDODEN and DO differ in the granularity. As Fig. 2 presents, for the “bearer of” relationship, no such relationship is observed in DO; three sub-relationships are observed in IDODEN; and four sub-relationships are observed in IDO, including “has disposition,” “has function,” “has quality” and “has role.” Since semantic granularity addresses the different levels of specification of an entity, we can conclude that IDO has the finest relative granularity according to the statistics and example.

**Fig. 3:**
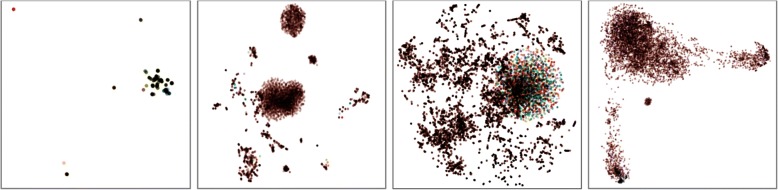
Ontology visualization for the node representations of IDO, IDODEN, DO and IDDAP. From left to right: the redundancy increases due to the structure sparseness, relation incompleteness, and unclear node definition

Ontology visualization is adopted to intuitively reflect the density of ontology information. The vertex classification experiment consists of comparing the two-dimensional visualization created from the embedding. We employ the network embedding projector (t-SNE) to further visualize the low-dimensional node representations learned by the embedding models. The node representations of four ontologies (IDO, IDODEN, DO and IDDAP) are presented in Fig. 3.

In Fig. 3(a), we can see that IDO ontology is constituted by 51 type of colors which indicate the connected components. Its simple structure and clear node definition leads to the lowest entropy value. The IDODEN and IDDAP ontology, although with complicated structure, is only composed by 27 and 19 types of colors respectively (Fig. 3(b) and (d)). The limited valuable information and loose structure of these two ontologies result in a certain degree of redundancy. In the DO ontology (Fig. 3(c)), there is 81 types of colors, which means this ontology contain the most connected components. The DO ontology is discrete, sparse, and unevenly distributed. There is overlap between the nodes, indicating that some of the information is redundant. The unwanted redundancy can be reduced or eliminated by data compression [24].

### Entropy evaluation performance

As ontological redundancy is mainly manifested in loose structures and lengthy textual information, we conducted experiments based on two aspects: ontologies with different structures and different textual information; and ontologies with unchanged structures but different textual information. The textual attention visualization and ontology statistical information is used as a reference to evaluate the validity of the calculations.

#### Data preprocessing

To conduct text embedding, the class definition in ontology is considered the text description. For a DO class that has no property or other text description, class labels are used as literal queries to extract no more than 5 sentences from the Wikipedia terminology introduction as the text description. For classes with no text description matching from Wikipedia, we have built the lineage of classes by performing a bottom-up extraction that copies text descriptions from their superclass nodes. For a DO class that has neither text description nor superclass description, the label is adopted as the text description.

#### Impact of textual information

To verify the effectiveness of incorporating textual information into the EAPB architecture, we randomly choose one entity (dengue virus maturation) as well as its causal and inheritance relationship from IDODEN ontology for the experiment. Dengue virus maturation is a biological process about the disease.

**Fig. 4:**
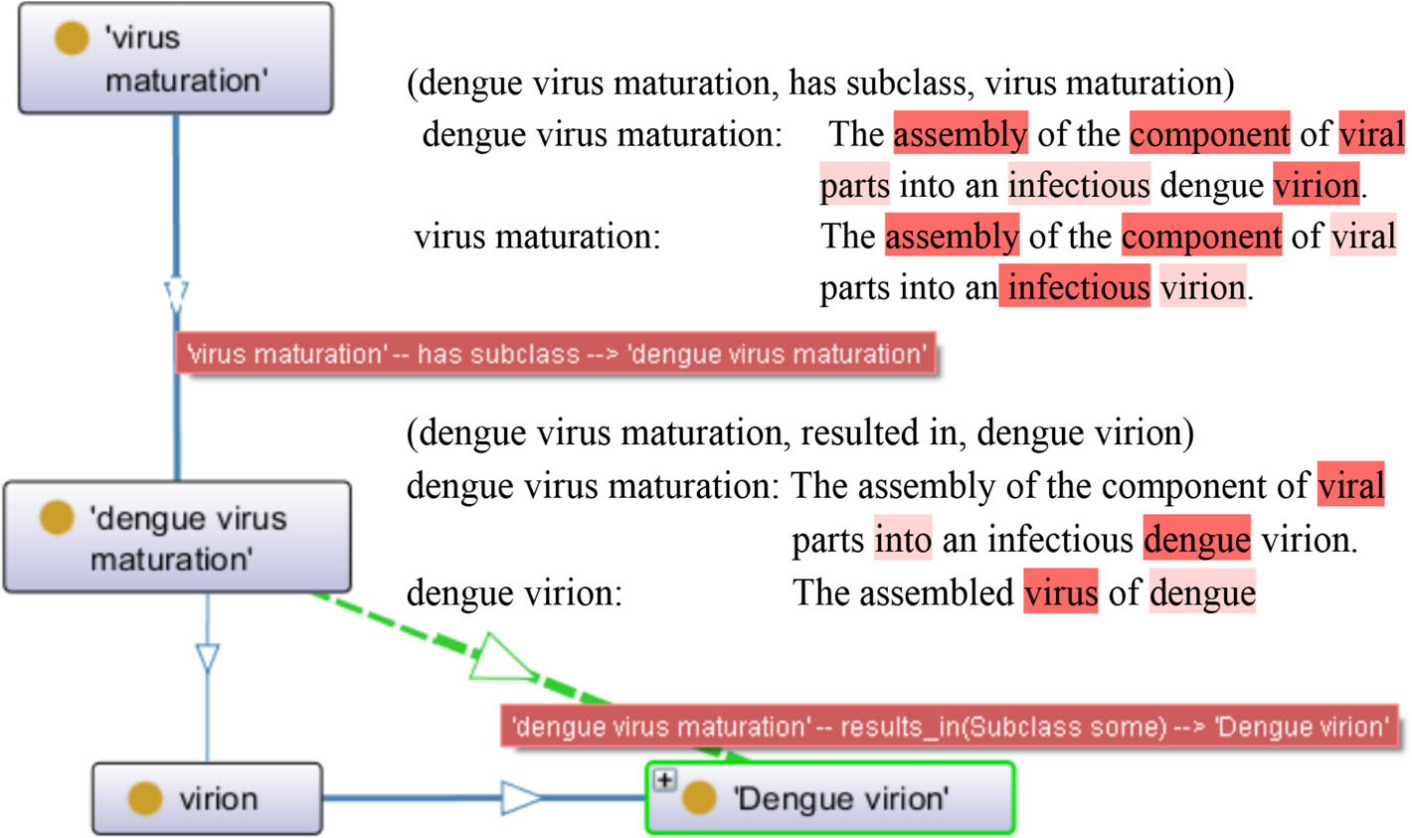
An example of the structural and textual information of IDODEN ontology. The higher the EAPB calculated information gain is, the more information the attention model highlights

It can be observed from Fig. 4 that, the class “dengue virus maturation” and “virus maturation” are directly connected, where the latter is a superclass of the former and their text information is thereby nearly the same. For another two indirectly connected classes, “dengue virus maturation” can cause “dengue virion”. Their descriptive text is different from each other. Consistently, the information gain between word pairs (dengue virus maturation, virus maturation) and (dengue virus maturation, dengue virion) is 6.7545 and 2.2228 respectively. This is within our expectation that the information gain between the former two classes is higher than that of the latter, which proves that the information gain obtained by our model can help to identify the textual information, so as to evaluate the ontology redundancy.

We visualize the attention scores predicted by EAPB in Fig. 4. The color depth indicates the importance degree of the words, the darker the more important. We can observe that, under the same ontology structure, a difference in text information contained in nodes will lead to differences in ontology redundancy; thus, the computation of entropy is useful in an ontology evaluation.

Furthermore, we employ four entire ontologies (IDO, IDODEN, DO and IDDAP) for the ablation tests of entropy evaluation in terms of discarding the textual information (w/o text). As one may expect, the consideration of textual information actually adjusts the network information density and affects the value of redundancy (see Table 2).

**Table 2:** Entropy evaluation result on IDO, IDODEN, DO and IDDAP (with ablation study)

	IDO	IDODEN	DO	IDDAP
EAPB entropy evaluation	5.0554	4.4507	8.2320	10.4234
w/o text	5.7469	8.4408	9.0656	14.5873

#### Performance comparison

According to the result reported in Table 2, Pearson correlation coefficient and Spearman rank correlation coefficient are adopted to evaluate the model performance. The IDO ontology, which is a well-recognized formal ontology for human infectious disease, is used as a base when calculating Pearson and Spearman rank correlation coefficients. Three other ontologies are compared with IDO from three aspects: the number of triples, the number of classes and entities, and the number of relations. The comparison results in three scores ranging from 0 to 1, obtained using the Softmax normalization method. To reduce the bias, we consider the average of these three scores as the final score. The geometric mean is employed to calculate the average, that is, the greater the difference, the smaller the mean; and the smaller the difference, the greater the mean.

Three aforementioned state-of-the-art baselines [5–7] are adopted for comparison (see Table 3). The experimental results on four ontologies are summarized in Table 3.

**Table 3:** Performance comparison result on IDO, IDODEN, DO and IDDAP

	Calmet [6]	Doran [7]	Gurupur [5]	EAPB
Entropy evaluation - Pearson coefficient				
IDO	0.1791	0.2352	0.2488	*0.3528*
IDODEN	0.2229	0.2494	0.2862	*0.5973*
DO	0.3387	0.3620	0.3962	*0.6857*
IDDAP	0.3729	0.3936	0.4215	*0.7121*
Entropy evaluation - Spearman rank correlation				
IDO	0.1727	0.2121	0.2131	*0.2913*
IDODEN	0.2254	0.2367	0.2581	*0.5257*
DO	0.3173	0.3616	0.3844	*0.5793*
IDDAP	0.3238	0.3857	0.4023	*0.6231*

According to the entropy evaluation result, the Pearson and Spearman rank correlation coefficients indicate that the EAPB is strongly correlated with the ontology statistical information (the numbers of triples, classes and entities, and relations summarized in Table 1) and Ontology visualization (see Fig. 3).

The result also shows that the IDDAP ontology scores obtained by calculating Pearson and Spearman rank correlation coefficients are higher than that of other ontologies. Although IDDAP has the largest numbers of triplets and classes/entities, it has the smallest number of relationships. Therefore, its loose structure and unevenly distributed nodes result in the highest redundancy. The IDODEN and DO ontologies have more numbers of triplets and classes/entities than IDO, leading to a certain degree of redundancy. The results of the performance comparison further validate the importance of evaluating the information redundancy by considering both structural information and textual information.

## Discussion and conclusion

At the technical level, we proposed a quality metric to assess the ontology entropy with information gain processed by connectivity matrix. Ontology can be considered as a graph or a network, the entropy rate of which is a measure of the complexity of the graph. Graph connectivity is generally used in graph entropy calculations; connectivity depending directly on the single point connectivity and equal path weight is considered as the weakest measure of network connectivity. Compared with existing measures of network connectivity, we defined a meaningful measure for connectivity that considers both connectivity path of the whole network and dynamic weight between different nodes. The experimental results proved that the EAPB could effectively evaluate ontologies, especially ontologies in the medical informatics field.

At the application level, the EAPB provides possible evaluation indicators for ontology engineers. We applied this new approach to evaluate an ontology that we developed as well as several well-known infectious disease-relevant ontologies including IDO, IDODEN, DO. The knowledge representation and information assessment do not simply “look up” structure or text but rather offer a reproducible “process” to assess the ontology by considering the number of node connections, the relationship between nodes, and the textual information included in the path.

Several points require further investigation in the next phase of research. First, the SST errors will be considered as one of the ontology quality evaluation metrics. SST errors can be used to relieve the problem of scalability because convergence significantly degrades as the network size increases [25]. Second, we will attempt to unify DeepWalk, LINE, and node2vec as a matrix factorization [26]. These network embedding algorithms are considered structure-only methods. However, their unification can address the problem of the relationship between the word-context matrix and the network. Third, we will explore ontology structure evaluation through normalized graph Laplacian, which is closer to the graph theory of ontology. Moreover, besides the exploration of asserted knowledge in the ontology, we will exploit the inferred knowledge from the asserted descriptions and compare their result differences to further improve our model.

## Methodology

This study proposes a metric for ontology quality that utilizes information divergence by considering both text-based and structure-based embeddings. Specifically, we first learn structure-based embeddings via a similar fashion with Node2vec, and learn the text-based embeddings via a CNN model with mutual attention [27], meanwhile, we conduct the optimization jointly to encode both path and text information into same representation space. Then we concatenate structure-based and text-based embeddings as vertices embeddings. Finally, the connectivity matrix of entropy computation is adjusted using the information gain obtained by the vertices embeddings.

### Structure-based embedding

The graph structure information is encoded by maximizing the log-likelihood of all directed edges. The structure-based energy function is given by:

1 $$ {L}_s(e)=\mathit{\log}p\left({v}^s|{u}^s\right) $$

As with Node2vec, we calculate conditional probability of vertices *v* generated by vertices *u* as:

2 $$ p\left({v}^s|{u}^s\right)=\frac{\mathit{\exp}\left({u}^s\bullet {v}^s\right)}{\sum_{k\in V}\mathit{\exp}\left({u}^s\bullet {k}^s\right)} $$

### Text-based embedding

**Fig. 5:**
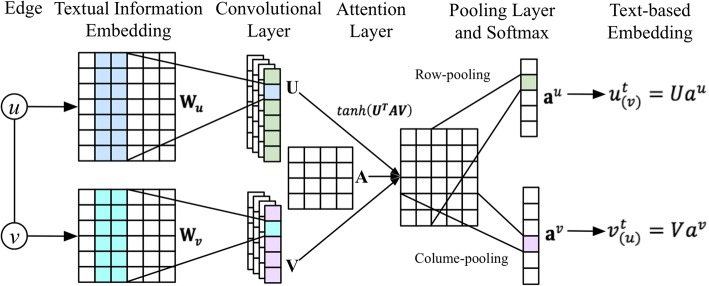
An illustration of text-based embedding

Given the word sequence of a vertex, we adopt CNN to capture the text information included in the ontology. The illustration of text-based embedding is presented in Fig. 5.

- **Input representation:** We use distributed word representation and transform sentence *S* = {*w*_1_, *w*_2_, …, *w*_*n*_} into corresponding word embedding sequence **W** = {**w**_**1**_, **w**_**2**_, …**w**_**n**_ } as input of CNN, where **w**_**i**_ ∈ ℝ^*d*^, *d* is dimension of the word embeddings.
- **Convolution:** For connected edge *e*_*u,v*_ with vertices *u* and *v*, we perform convolution operation over a sliding window to extract local features of their textual embeddings **W**_***u***_ and **W**_***v***_, where **W**_***u***_ ∈ *ℝ*^*d* ∗ *m*^, **W**_***v***_ ∈ ℝ^*d* ∗ *n*^, *m* and *n* represents the lengths of **W**_***u***_ and **W**_***v***_ respectively.
- **Attentive Pooling:** To encode the vertices interactive information facing different neighbors, we apply mutual attention into the pooling layer. To be specific, based on the output of convolution layer **U** and **V** respectively for vertice *u* and *v*, we introduce an attentive matrix *A* ∈ ℝ^*d* ∗ *d*^ and calculate the correlation matrix C ∈ ℝ^*m* ∗ *n*^, which represents the pair-wise correlation score between U and V, as follow:

3 $$ C=\mathit{\tanh}\left({\boldsymbol{U}}^{\boldsymbol{T}}\boldsymbol{AV}\right) $$

Intuitively, the attentive matrix is used to assign different weights according to the specific role each vertex plays when interacting with other vertices. Then we conduct mean pooling operations along the rows and columns of C to generate the row-pooling and column-pooling respectively:

4 $$ {\displaystyle \begin{array}{l}{h}_i^u= mean\left({C}_{i,1},\dots, {C}_{i,n}\right)\\ {}{h}_i^v= mean\left({C}_{1,i},\dots, {C}_{m,i}\right)\end{array}} $$

where ${h}_i^u$ and ${h}_i^v$ indicate importance score for word *i* when interacting with vertice *v* and *u* respectively. Next we obtain attention vectors **a**^*u*^ and **a**^*v*^ from ${\mathbf{h}}^u=\left[{h}_1^u,\dots, {h}_m^u\right]\hat{\mkern6mu} T$ and ${\mathbf{h}}^v=\left[{h}_1^v,\dots, {h}_n^v\right]\hat{\mkern6mu} T$ by employing softmax function.

Finally, the text-based embeddings of *u* and *v* are calculated as:

5 $$ {\displaystyle \begin{array}{l}{u}_{(v)}^t={Ua}^u\\ {}{v}_{(u)}^t={Va}^v\end{array}} $$

### Optimization

We learn the text-based and structure-based representations by maximizing their energy function jointly as:

6 $$ \mathcal{L}=\sum \limits_{e\in E}{L}_t(e)+{L}_s(e) $$

where *E* indicates all edges of learned ontology, *L*_*s*_(*e*) is structure-based energy function in Eq. (1). As for text-based energy function L_t_(e), we aim to map two types of vertex embeddings into the same representation space and define it as:

7 $$ {L}_t(e)=\alpha \cdot {L}_{tt}(e)+\beta \cdot {L}_{ts}(e)+\gamma \cdot {L}_{st}(e), $$

8 $$ {\displaystyle \begin{array}{l}{L}_{tt}(e)=\mathit{\log}\ p\left({v}^t|{u}^t\right),\\ {}{L}_{ts}(e)=\mathit{\log}\;p\left({v}^t|{u}^s\right),\\ {}{L}_t{L}_{st}(e)=\mathit{\log}\;p\left({v}^s\left|{u}^t\right.\right)\end{array}} $$

where *α*, *β* and *γ* are used to control the weights of *L*_*ts*_(*e*), *L*_*st*_(*e*) and *L*_*tt*_(*e*) , the former two indicates mutual generation based on text and structure, meanwhile, we expect them can contain their own characteristics by *L*_*tt*_(*e*). All of them are adopted similar softmax computation as structure-based as Eq. (1).

### Weighted connectivity matrix

Entropy can be calculated via the network probability distribution function. The connectivity matrix Cn*n is dedicated to calculating node probabilities, where *n* stands for the number of nodes in the ontology. The connectivity matrix includes the connection information between *u* and *v*, where 0 demonstrates no connection between *u* and *v* and 1 indicates a connection. The probabilities *P*_*k*_ of each concept *k* can be processed by:

9 $$ {P}_k=\frac{\sum_{u=1}^n{C}_{u\mathrm{k}}}{\sum_{v=1}^n{\sum}_{u=1}^n{C}_{uv}} $$

Given the structure-based and text-based embedding concatenation results, the relevancy between nodes *u* and *v* can be evaluated using a cosine similarity measure. When nodes *u* and *v* are not directly connected, the selected path is their shortest path. To avoid multiple calculations of the same path’s weight, the relevancy is divided by the shortest path, the result of which is considered to be the information gain and represented as *O*_*uv*_. We multiply *O*_*uv*_ by the connectivity matrix *C*_*uv*_ of the entropy computation. As a result, the probabilities *P*_*k*_ of each concept *k* can be given by:

11 $$ {P}_k^{\prime }=\frac{\sum_{k\ne u}{\sum}_{u=1}^n{C}_{uk}\ast {O}_{uk}}{\sum_{v=1}^n{\sum}_{u=1}^n{C}_{uv}\ast {O}_{uv}} $$

The path-based text-aware calculation formula for determining entropy using diverse node weights can be presented as:

12 $$ {\mathrm{S}}^{\prime }=-{\sum}_{k=1}^n{P}_k^{\prime }{\log}_2{P}_k^{\prime } $$

## Abbreviations

DO: Disease ontology
EAPB: Entropy-Aware Path-Based metric for ontology quality
IDDAP: Ontology-driven clinical decision support system for infectious disease diagnosis and antibiotic prescription
IDO: Infectious disease ontology
IDODEN: Infectious disease ontology for dengue
NLM: The United States national library of medicine

## Footnotes

1
http://infectiousdiseaseontology.org/page/Main_Page

2
https://code.google.com/archive/p/dengue-fever-ontology/

3 doi: 10.1093/nar/gku1011

4
www.obofoundry.org/ontology/ncbitaxon.html

5 human-phenotype-ontology.github.io/

6 https://www.drugbank.ca/

7
http://www.hopkinsmedicine.org/amp/guidelines/antibiotic_guidelines.pdf

8 https://www.nlm.nih.gov/
